# General practice-related MeSH terms in main journals: a bibliometric analysis from 2011 to 2021

**DOI:** 10.3399/BJGP.2023.0060

**Published:** 2024-01-23

**Authors:** Théo Duguet, Gladys Ibanez, Matthieu Schuers, Jean-Pierre Lebeau, Kristell Roser, Cécilia Saldanha Gomes, Jean-Sébastien Cadwallader

**Affiliations:** Department of General Practice, Sorbonne University, Paris.; Department of General Practice, Sorbonne University, Paris, and INSERM, Sorbonne University, Institut Pierre Louis d’Épidémiologie et de Santé Publique (IPLESP), Paris.; Department of General Medicine, Rouen University Hospital, Rouen; Department of Biomedical Informatics, CHU Rouen, Rouen, and INSERM U 1142, LIMICS.; Department of General Practice, University of Tours, Tours.; Inter-University Health Library, Medicine-Odontology Center, Paris.; Department of General Practice, Sorbonne University, Paris, and INSERM, Sorbonne University, Institut Pierre Louis d’Épidémiologie et de Santé Publique (IPLESP), Paris.; Department of General Practice, Sorbonne University, Paris, and INSERM, Sorbonne University, Institut Pierre Louis d’Épidémiologie et de Santé Publique (IPLESP), Paris.

**Keywords:** bibliometrics, family physicians, primary care, family practice, general practice, general practitioners, physicians, primary health care

## Abstract

**Background:**

There are various Medical Subject Headings (MeSH) terms used to index general practice research, without consistency.

**Aim:**

To understand how general practice-related research is indexed in the main general practice journals between 2011 and 2021, and to analyse the factors that influenced the choice of the general practice-related MeSH.

**Design and setting:**

This was a quantitative bibliometric study conducted on MEDLINE.

**Method:**

MeSH were selected according to the international definition of General Practice/Family Medicine: ‘General Practice’, ‘Primary Health Care’, ‘Family Practice’, ‘General Practitioners’, ‘Physicians, Primary Care’, and ‘Physicians, Family’. Their use was studied from 2011 to 2021 on MEDLINE, reviewing the 20 general practice journals with the highest impact factors. A descriptive and analytical approach was used; the association of the country, journal, and year with the choice of general practice-related MeSH terms was analysed.

**Results:**

A total of 8514 of 150 286 articles (5.7%) were using one of the general practice-related MeSH terms. The most used were ‘Primary Health Care’ (4648/9984, 46.6%) and ‘General Practice’ (2841/9984, 28.5%). A total of 80.0% (6172/7723) of the articles were related to the UK or US and 71.0% (6055/8514) of the articles came from four journals (*BJGP*, *BMJ*, *Journal of General Internal Medicine*, and *Annals of Family Medicine*). Two main country clusters emerged from the use of general practice-related MeSH: a British cluster mainly using ‘General Practice’ and an American cluster using ‘Primary Health Care’. The journals also mainly differed in their used of these two MeSH terms.

**Conclusion:**

Important variations in the indexation of general practice research were found. Researchers should consider combining ‘Primary Health Care’ and ‘General Practice’ in their PubMed searches to access all the general practice research, regardless of their country of origin.

## Introduction

Research is fundamental to improving health care. Primary healthcare-/general practice-centred research is growing every year.^1^^,^^2^ There are important differences between countries concerning their contribution to general practice-related research. The UK and the US are the top publishers, followed by other English-speaking countries.^1^^,^^3^ The UK and the US have the top researchers according to a study published in 2011,^4^ the most cited articles,^5^ and the highest number of publications by population.^6^ Many countries, especially in Europe, are developing general practice-centred research and trying to close the gap.^7^^–^9

Primary care research is growing and gaining recognition, as shown by the recent creation of a specific index for primary care journals in the Journal Citation Index within the Science Citation Index (SCI), with journals from various countries.^10^^,^^11^ Most of this research is indexed on worldwide databases such as MEDLINE that makes it possible to consult a large amount of general practice-related research.^12^ MEDLINE is organised with about 30 000 Medical Subject Headings (MeSH) used as descriptors, with an annual update of the list. Most medical specialties have one or two MeSH terms as descriptors. When articles are included in the MEDLINE database, they are assigned MeSH terms. MeSH assignments, previously carried out by human National Library of Medicine (NLM) workers and gradually replaced by an automatic algorithm, follow indexing rules based on the vocabulary used in the title and abstract. The keywords section is not used by this algorithm for the MeSH choice.

The differences between health systems, however, have led to varied nomenclature to describe general practice, with geographical areas where general practice is the main terminology (as in the UK) and others where family medicine and primary health care are the main terminologies (as in the US).

**Table 1:** How this fits in

The Medical Subject Headings (MeSH) system, an essential tool in PubMed, indexes research related to general practice with various terms because of the history and diversity of healthcare systems (for example, ‘Primary Health Care’ or ‘General Practice’). This study aimed to investigate the association between general practice-related MeSH terms in the most influential peer-reviewed journals and to understand the association of the countries, journals, and publication year with the choice of general practice-related MeSH terms. This research will allow researchers using PubMed or carrying out meta-analyses to access comprehensive knowledge in general medicine, transcending the origins and sources of publication.

Nowadays, even when international organisations are trying to unify the terms, especially between general practice and family practice,^13^ different names remain in the MeSH thesaurus: ‘General Practice’, ‘Primary Health Care’, ‘Family Practice’, ‘General Practitioners’, ‘Physicians, Primary Care’, and ‘Physicians, Family’, reflecting these geographical specificities with distinct but overlapping definitions (Supplementary Information S1). Since 2011, ‘Family Practice’ has been included as a narrower term under ‘General Practice’, which seems to be an encouraging sign of the homogenisation of terminologies.

Increasing the visibility of general practice-related research is an important issue, both for the general public and the medical community.^14^ To be able to disseminate innovation in general practice to the medical community, it is important to have easy and clear referencing of the studies. This is necessary both for physicians who need to access the latest data and for researchers who need to make their research as visible as possible. The diversity of general practice-related MeSH terms may complicate the visibility of these articles and lead to the risk of lost knowledge for the entire scientific community.

To the authors’ knowledge, no study has analysed how general practice articles have been referenced in the main journals and what influence the choice of the general practice-related MeSH terms has had. The objective of the current study was to understand how general practice-related research is indexed in the main general practice journals and to analyse the factors associated with the choice of the general practice-related MeSH terms in these journals between 2011 and 2021.

## Method

A quantitative bibliometric study of the MEDLINE database was undertaken. General practice-related MeSH terms were selected according to their definition as given by the NLM. In the current study, terms were kept if their definition corresponded to the international definition of general practice/family medicine according to the WONCA-EURACT,^13^ these MeSH terms were ‘General Practice’, ‘Primary Health Care’, ‘Family Practice’, ‘General Practitioners’, ‘Physicians, Primary Care’, and ‘Physicians, Family’.

The period of interest extended from 1 January 2011 to 31 December 2021. In order to study the current general practice-related MeSH terms, the starting point was the last time the general practice-related MeSH terms list was modified (2011). At this date ‘General Practice’, ‘General Practitioners’, and ‘Physicians, Primary Care’ were added.

To define the list of journals to analyse, the authors started by including all the journals indexed in the SCI database with ‘ML’ – Primary Health Care or ‘PY’ – Medicine General and Internal. As the aim was to analyse the most influential research worldwide, journals with the highest impact factor were selected. Although the impact factor is heavily criticised for several biases inherent in its calculation,^15^ it is still used in many institutions around the world as a means of funding research and researchers, or for researchers’ careers, particularly at the time of the current study.^16^^,^^17^ Given these considerations, the most cutting-edge research is more likely to be published in journals with a high impact factor and is therefore more likely to be read by a larger number of readers. In the current study, the authors defined the most influential journals with an impact factor threshold of five. The threshold of five is arbitrary and was considered to provide a smaller but analysable number of journals and articles.

In addition, a sensitivity analysis was conducted using a different list of journals. For this sensitivity analysis, all journals indexed under the category ‘Primary Health Care’ in the SCI were included without considering their impact factors.

For the main analysis, journals with <20 occurrences of general practice-related MeSH terms during the period of interest were excluded. The result was a list of 20 influential general practice journals. A flow chart and list of journals are available in Supplementary Figure S1. During this period and in these journals, 150 286 articles were extracted. Then those that included at least one of the current study’s chosen general practice-related MeSH terms were selected; 8514 articles. Any document defined on MEDLINE as a single journal article with the minimal use of a MeSH term was analysed as an article.

First, a descriptive analysis of these 8514 articles was undertaken, with the proportion of each general practice-related MeSH term and the contribution of each country, journal, and year added to the study database. For each article, the first author’s address was used to localise the article. When this information was missing and if a country was mentioned in the MeSH terms’ list, this information was used to localise the article. The authors of the current study only analysed countries with a minimum of 10 localised MeSH terms

Next, comparative analyses were carried out, examining the factors associated with the choice of the different general practice-related MeSH terms, specifically the country, journal, and year. For these analyses, the authors used comparative histograms and explorative models with a hierarchical clustering on principal components (HCPC). For the HCPC, each country was considered as an individual, and the analysis took into account the proportion of use of each general practice-related MeSH term for the different countries. The software R version 4.1.2 was used.

## Results

### Descriptive approach

In total, 18.0% (1533/8514) of the articles did not provide the first author’s address. It was possible to localise 91.8% of the articles.

In the 20 main general practice-related journals selected, 90.7% of articles (7723/8514) used at least one of the general practice-related MeSH terms. In these 8514 articles, there were 9984 occurrences of general practice-related MeSH terms (some of the articles using more than one general practice-related MeSH term).

The most used general practice-related MeSH term was ‘Primary Health Care’ (46.6%, 4648/9984), followed by ‘General Practice’ (28.5%, 2841/9984). The other MeSH terms by frequency were ‘Family Practice’ (9.7%, 964/9984), ‘General Practitioners’ (8.7%, 869/9984), ‘Physicians, Primary Care’ (4.7%, 465/9984), and ‘Physicians, Family’ (2.0%, 197/9984).

**Figure 1.**
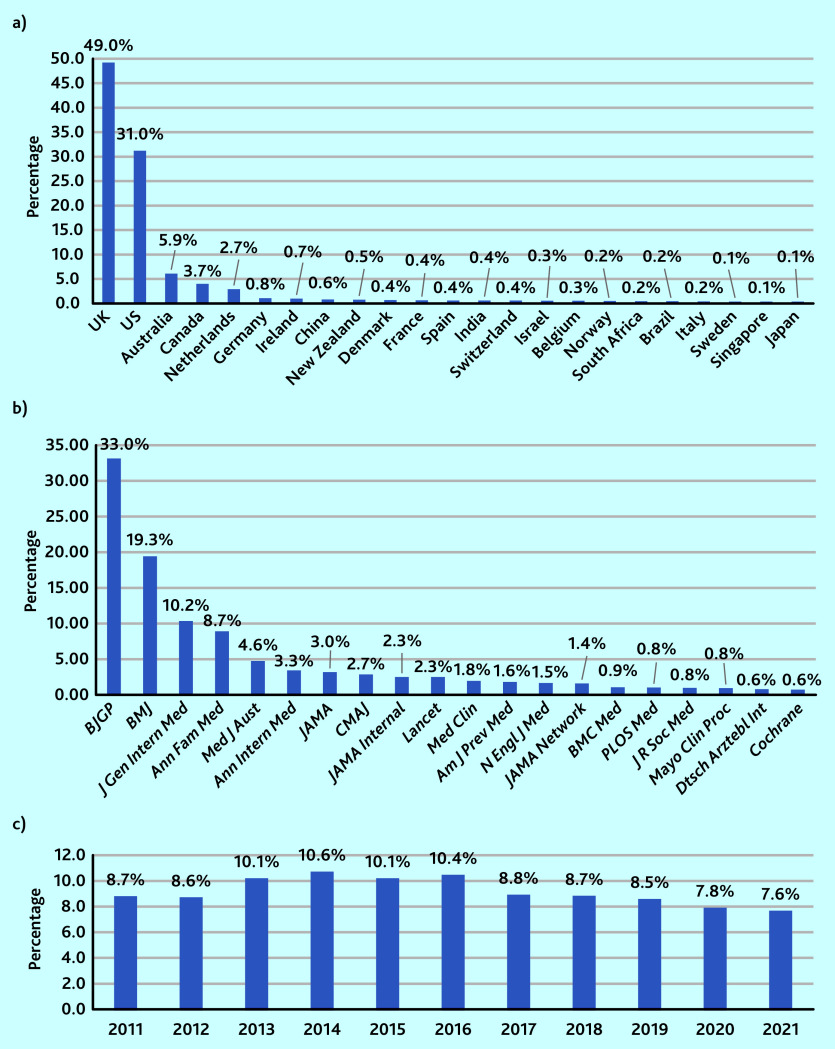
*Proportion of articles using at least one general practice-related MeSH term by a) country, b) journal (using National Library of Medicine title abbreviations), and c) year,* n *= 8514. MeSH = Medical Subject Headings.*

When the articles were analysed according to the first author’s country, a list of 65 countries was obtained (Supplementary Table S1). Countries contributed in a widely varied way to the selected 8514 articles, with 80.0% (6172/7723) of these articles being linked to the UK and US (Figure 1a).

When looking at the journals, 71.0% (6055) of the 8514 articles came from only four of the 20 journals (*BJGP*, *BMJ*, *Journal of General Internal Medicine*, and *Annals of Family Medicine*) (Figure 1b). In two of the journals (the *BJGP* and *Annals of Family Medicine*), more than 60% (*BJGP* 2818/4601; *Ann Fam Med* 745/1207) of articles used at least one of the general practice-related MeSH terms, whereas 13 of the journals used them in <5% of their articles (data available on request from the authors).

There were fewer variations relating to the year of publication. Of the 8514 articles there was a nadir of articles in 2021 (7.6%, 645/8514) and a zenith in 2014 (10.6%, 904/8514) (Figure 1c).

### Associated factors for the use of general practice-related MeSH terms

#### Country of publication

**Figure 2.**
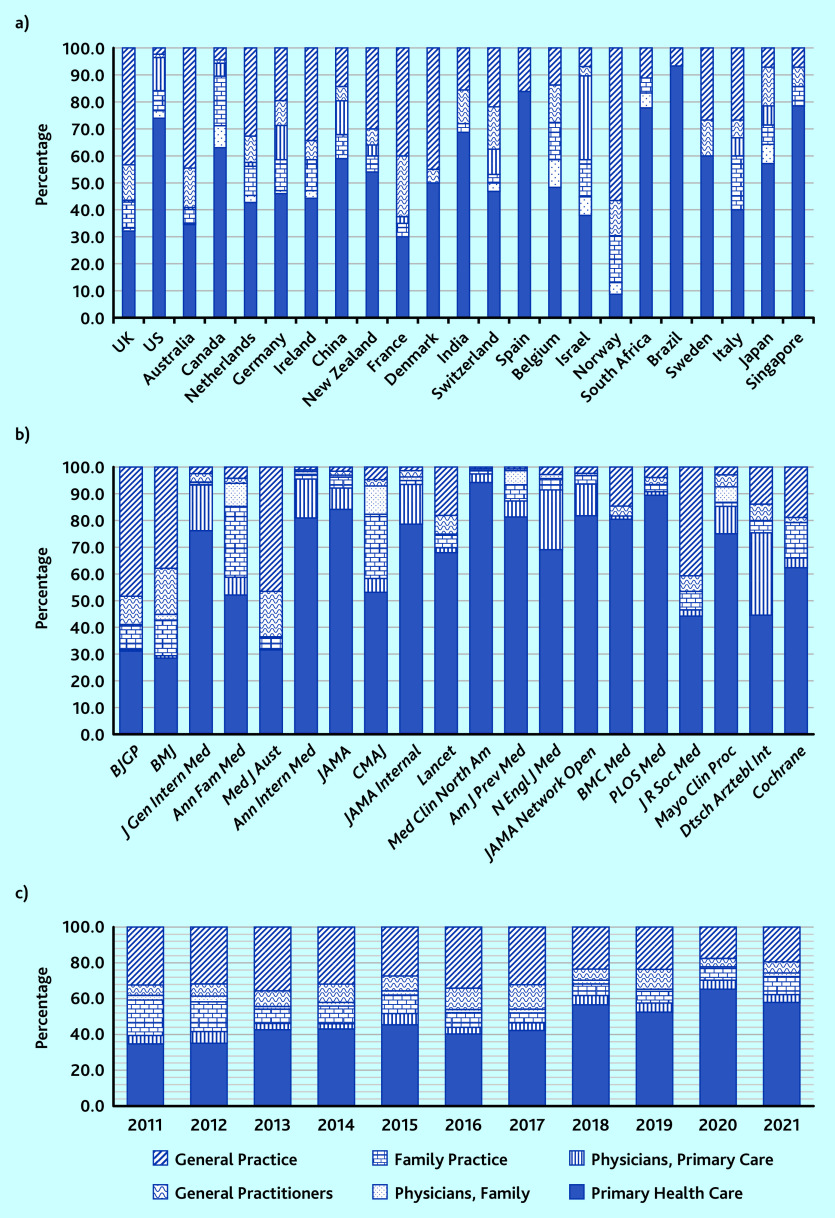
*Proportional use of general practice-related MeSH terms according to a) country, b) journal (using National Library of Medicine title abbreviations), and c) year,* n *= 9984. JAMA Network = JAMA Network Open. Med Clin = Med Clin North Am. MeSH = Medical Subject Headings.*

Looking at the use of general practice-related MeSH terms according to the country of the first author, some countries were using ‘General Practice’ as a main MeSH term such as the UK, Australia, and France, and others were using ‘Primary Health Care’ as a main MeSH term such as the US and Canada (Figure 2a).

**Figure 3.**
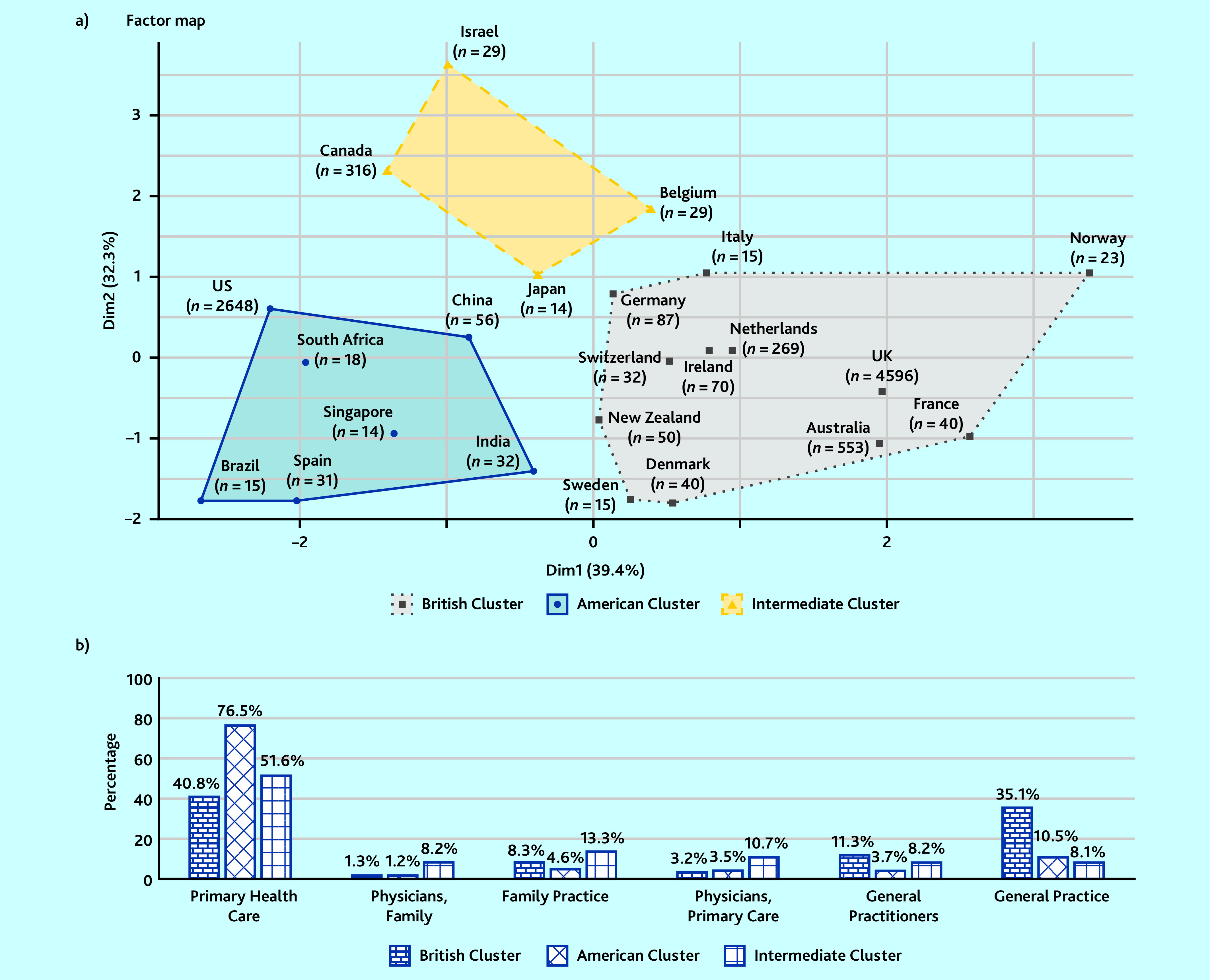
*Country clusters from HCPC with publication profiles (‘*n*’ is the number of articles from each country). a) Hierarchical clustering on principal component analysis of the different countries considering the proportion of use of the six general practice-related MeSH terms for each country. The axis corresponds to the two principal components with their fraction of the total variance in parentheses. b) Proportion of use of the different MeSH terms in each country cluster made from the HCPC. Dim = dimension. HCPC = hierarchical clustering on principal components. MeSH = Medical Subject Headings.*

According to the HCPC, two main country clusters emerged from the way general practice-related MeSH terms were used: a ‘British cluster’ composed of 12 countries and an ‘American cluster’ composed of seven countries (Figure 3a). The British cluster accounted for 64% (5790/9103) of all the localised MeSH terms against 31% (2814/9103) for the American cluster. A third, smaller cluster was identified, with only 4% (388/9103) of the localised MeSH terms. Looking at each cluster, homogeneous indexing methods could be seen inside each cluster and there were differences between the different clusters (Figure 3b).

#### Journal

Some of the journals were predominantly using ‘General Practice’ such as the *BJGP* or the *BMJ*, whereas others were predominantly using ‘Primary Health Care’ such as *Annals of Family Medicine*, *Annals of Internal Medicin*e (Figure 2b). Furthermore, all journals from North America were using ‘General Practice’ <10% of the time, whereas all journals from outside of North America were using this MeSH term >10% of the time (up to 48.4% for the *BJGP*, 1723/3560) (Supplementary Table S2).

#### Year of publication

According to year of publication, it could be seen that the use of ‘General Practice’ was decreasing in proportion, from 34.1% (355/1042) in 2016 to 19.4% (153/787) in 2021 (Figure 2c).

## Discussion

### Summary

The proportion of articles using general practice-related MeSH terms was low. Even though the journals were only selected if they were indexed as general practice-related on the Web of Science, very few used a significant proportion of general practice-related MeSH terms. The MeSH term ‘Primary Health Care’ was the most used, followed by ‘General Practice’. The UK and the US were the source of an overwhelming majority of the articles. The proportion of articles seemed to slightly decrease over the period. Few articles were using both ‘General Practice’ and ‘Primary Health Care’, 5.2% (445/8514) mainly coming from the UK 72.1% (310/430).

The first author’s country had a major association with the general practice-related MeSH terms used. The countries divided into two main clusters. The countries composing the ‘British cluster’ mainly used ‘General Practice’, with this term used only rarely in the ‘American cluster’ where ‘Primary Health Care’ was largely predominant. The term ‘General Practice’ was very close to the European definitions of general practice^10^ but the shown association between countries and its use seem to be a brake on the homogenisation of the MeSH term use. The journal was also associated with the choice of general practice-related MeSH terms, but in the current study the authors were unable to identify global patterns. The year of publication seemed to be less associated with the use of general practice-related MeSH terms.

### Strengths and limitations

This study is, to the authors’ knowledge, the first one to analyse the association of country, journal, and year, and the choice of general practice-related MeSH terms. The results of this study, which show geographical patterns of use of MeSH terms, are consistent with the known variation in regional terminology for general practices and primary care.

The main limitation of this study was its focus on the most influential general practice journals on a global scale based on the impact factor and with exclusion of journals with a lower impact factor. This strategy excluded most of the regional journals that can have a significant influence at a local level. However, even with these criteria, the countries publishing the most articles that have been identified in the current study are the same as in previous studies.^1^^,^^3^ And the results of the sensitivity analysis were consistent with the main findings (data available on request from the corresponding author). The use of other indicators such as the Eigenfactor could also have been used to strengthen the choice of journals. A new study without a limitation on journals should be conducted to confirm the current findings.

Another limitation to consider is that indexers (human or machine) are encouraged to index at the highest level of specificity. Discipline MeSH terms, if they do not correspond to the subject of the article, may therefore not be retained in favour of more specific MeSH terms. For example, an article about preventing a specific disease in general practice might be indexed only under the disease and the prevention-related MeSH terms. This point explains the low rate of general practice-related MeSH terms in general practice journals. The current study focused on the use of discipline MeSH terms (‘General Practice’, ‘Primary Health Care’, ‘Family Practice’). However, it does not allow a clear conclusion to be drawn on the criteria for preferring discipline terms over specialist-related terms (‘General Practitioners’, ‘Physicians, Primary Care’, ‘Physicians, Family’).

Another limitation arises from the potential for certain journals, characterised by a significant volume of publications, to bias countries’ patterns and excessively highlight countries or MeSH terms. To address this issue, further research should be undertaken to analyse this aspect.

Finally, the authors of the current study only used a bibliometric approach, and MeSH terms are chosen by the NLM after analysis of the vocabulary used by the authors in the title and abstract, and are therefore indirect markers. An individual comparison of the studies would be useful to be sure that MeSH differences were not explained by internal differences of topic and contents between studies. In addition, an analysis of the association between MeSH terms and the author’s vocabulary could confirm the fidelity of the NLM indexing method.

### Comparison with existing literature

This article is, to the authors’ knowledge, the first one to explore this aspect of MeSH use. Nevertheless, the countries publishing the most articles that have been identified in the current study are the same as in previous studies.^1^^,^^3^

### Implications for research

General practice has common ground between countries and is globally recognised as a cornerstone of sustainable healthcare systems.^18^ Thus, it is important to promote primary care research. Using MeSH terms in the PubMed browser is a very effective way of finding relevant searches. In the specific field of general practice-related research, knowledge of the geographical patterns of MeSH terms seems to be a useful and easy-to-use tool to promote access to research.

Based on the findings in the current study, the authors therefore suggest that the use of the combination of both MeSH terms ‘Primary Health Care’ and ‘General Practice’ should be considered by researchers in their PubMed searches. In addition, the authors of the current study encourage authors to use their discipline words such as ‘General Practice’ or ‘Primary Health Care’ in their titles and abstracts to increase the chances of being indexed with general practice-related MeSH terms. This consistency would make primary care research more visible around the world and thus improve quality of care for all.

## Data

All data used in this article are open access and can be freely extracted from PubMed.
